# DeepOSSA: a deep learning framework for personalized survival prediction in osteosarcoma patients

**DOI:** 10.3389/fcell.2026.1868333

**Published:** 2026-10-22

**Authors:** Zongdong Zhu, Xiaowei Sha, Chengliang Yin, Ge Fan, Wei Lu

**Affiliations:** 1 Orthopedics, Sichuan Provincial People’s Hospital, School of Medicine, University of Electronic Science and Technology of China (UESTC), Chengdu, China; 2 Department of Dermatology, Sichuan Provincial People’s Hospital, School of Medicine, University of Electronic Science and Technology of China (UESTC), Chengdu, China; 3 Department of Biomedical Engineering, Faculty of Engineering, Universiti Malaya, Kuala Lumpur, Malaysia; 4 Independent Researcher, Shenzhen, China

**Keywords:** deep learning, osteosarcoma, sparse feature interaction, survival prediction, Transformer

## Abstract

**Background:**

Osteosarcoma, the most common primary malignant bone tumor in children and adolescents, is marked by high invasiveness and heterogeneous outcomes due to inter-patient variability in tumor biology and treatment response. Accurate prognostic modeling is crucial for personalized risk stratification and clinical decision-making.

**Objective:**

We developed and validated DeepOSSA, a deep learning framework for individualized survival prediction in osteosarcoma, leveraging high-dimensional, sparsely encoded clinical data from the Surveillance, Epidemiology, and End Results (SEER) database.

**Methods:**

Clinical data from 895 osteosarcoma patients diagnosed between 2010 and 2018 were extracted. Continuous variables were optimally discretized using X-tile, and categorical covariates were one-hot encoded. We compared DeepOSSA against seven representative models spanning classical machine learning (support vector machine (SVM), random forest (RF), and gradient boosted decision trees (GBDT)), deep learning (multi-layer perceptron (MLP) and Transformer), and survival analysis (Cox proportional hazards (CoxPH) and DeepSurv). DeepOSSA integrates a Transformer-based representation tower for high-order sparse feature crossing with a Cox-style discrete-time survival head, enabling joint optimization of feature representation and time-to-event modeling. Performance was evaluated on a held-out test set using the area under the curve (AUC) and F1-score.

**Results:**

DeepOSSA outperformed all baselines in discrimination and balanced classification, achieving an AUC of 0.8058±0.0081 and an F1-score of 0.6506±0.0060 , representing average relative improvements of 13.11% (AUC) and 8.99% (F1), respectively. Among all evaluated models, DeepOSSA achieved the highest AUC (exceeding 0.8) and demonstrated competitive performance compared with previously published, externally validated Cox nomograms for osteosarcoma. In time-dependent evaluation, its predictive advantage over CoxPH increased during long-term follow-up, where proportional hazards assumptions are most violated. Ablation studies confirmed the necessity of each architectural component, and SHapley Additive exPlanations (SHAP) analysis demonstrated that the model’s risk predictions are driven by clinically established prognostic factors. By capturing attention-based nonlinear interactions and temporal dynamics, DeepOSSA enables more precise patient stratification than traditional linear models.

**Conclusion:**

DeepOSSA is a robust and interpretable framework that bridges Transformer-based representation learning with principled survival analysis. It effectively models complex, nonlinear relationships among sparse clinical features and survival outcomes, offering strong potential for translating heterogeneous clinical data into actionable insights for precision oncology.

## 1 Introduction

Osteosarcoma (OSC) is a malignant bone tumor that arises from primitive mesenchymal cells and exhibits highly aggressive and heterogeneous biological behavior. Although the widespread adoption of multi-modal adjuvant chemotherapy and limb-salvage surgery over the past decades has substantially improved survival rates for patients with localized osteosarcoma, the prognosis for patients presenting with metastases at initial diagnosis, particularly pulmonary metastases, or with recurrent disease remains exceedingly poor, with the 5-year survival rate persistently stagnating between 20% and 30% (Korn et al., 2011; Siegel et al., 2023). The bimodal age distribution of osteosarcoma incidence, peaking during adolescence and again in older adults, further compounds the heterogeneity challenges faced in clinical decision-making and motivates the development of more refined, individualized prognostic tools.

In current clinical practice, physicians predominantly rely on the American Joint Committee on Cancer (AJCC) TNM staging system or the Musculoskeletal Tumor Society (MSTS) grading system for prognostic assessment (Greene et al., 2002; Cates, 2017). However, these conventional classification systems are based solely on a small set of morphological indicators such as tumor size, lymph node status, and distant metastasis, and are therefore insufficient to capture the complex nonlinear interactions among patient demographics, fine-grained pathological details, and therapeutic interventions. Furthermore, traditional statistical tools such as the Cox proportional hazards model (CoxPH) (Lin and Wei, 1989; Kumar and Klefsjö, 1994), while robust on small samples, are constrained by the strict proportional hazards assumption and the premise of linear and multiplicative covariate effects, which renders them inadequate for the high-dimensional, interaction-rich clinical data of the modern era (Li et al., 2023; Meng et al., 2025; Chokkakula et al., 2026; Jeyaraj et al., 2026).

With the rapid advancement of computational medicine and artificial intelligence, machine learning (ML) and deep learning (DL) have introduced new paradigms for addressing these challenges (Yang et al., 2018; Du et al., 2020; Xie et al., 2023; Zhang et al., 2025; Saraf et al., 2025; Fan et al., 2026). Such approaches, particularly deep neural networks, which learn latent patterns by passing inputs through successive hidden layers and adjusting connection weights during training, can extract latent patterns from large-scale, high-dimensional clinical data and thus enable more precise individualized risk stratification (Katzman et al., 2018; Wang et al., 2022; Lian et al., 2024). Nevertheless, existing approaches in the osteosarcoma domain still face two critical bottlenecks (Hao et al., 2023; Spreafico et al., 2024a; Zhao et al., 2025). First, clinical registry data are dominated by sparsely encoded categorical covariates, such as one-hot tumor stage, primary site, and metastasis indicators, whose prognostic information lies primarily in the high-order interactions among them; tree-based ensembles (e.g., random forest and XGBoost) (Chen et al., 2015; Biau and Scornet, 2016) and shallow MLPs treat these one-hot tokens as independent features and struggle to learn deep, all-pairs feature crossings. Second, general-purpose deep architectures such as plain MLPs and Transformers lack survival-specific inductive biases: they treat prognosis as a static classification target and ignore censored time-to-event structure, while existing survival neural networks such as DeepSurv inherit the proportional hazards assumption of CoxPH and therefore cannot fully exploit the representational power of deep models (Hu et al., 2021; Li et al., 2025).

To address these limitations, this study proposes Deep learning for OSteoSarcoma Survival Analysis (DeepOSSA), a deep survival prediction framework that explicitly couples a Transformer-based representation tower with a Cox-style discrete-time survival head. By tokenizing each one-hot encoded clinical variable and feeding the resulting token sequence into a stack of multi-head self-attention layers, DeepOSSA performs dense, all-pairs crossing of sparse categorical features and adaptively re-weights their interactions on a per-patient basis, producing high-level patient representations that linear models and tree ensembles cannot reach. These representations are then projected through a shallow Cox-style hazard head trained under a discrete-time negative log-likelihood objective, so that representation learning and time-to-event modeling are optimized jointly within a single network rather than decoupled into a feature extractor followed by an external Cox model. An auxiliary debiasing tower operating on the raw embeddings further mitigates distributional bias inherent in clinical registries such as SEER, complementing the deep tower with a residual signal that stabilizes prediction.

The main contributions of this article are summarized as follows:

1. Sparse feature crossing via attention. We design a Transformer-based representation tower that tokenizes one-hot encoded clinical variables and uses multi-head self-attention to perform dense, high-order crossing of sparse categorical features, capturing patient-specific interactions among demographics, tumor characteristics, metastatic status, and treatment that linear and tree-based survival models cannot capture.
2. Coupling Transformer representations with the Cox framework. We integrate the Transformer-based representation tower with a Cox-style discrete-time survival head and optimize the entire network end-to-end under a negative log-likelihood objective, combining the high-level representational capacity of attention-based deep models with the principled time-to-event modeling of the Cox framework.
3. Comprehensive empirical validation on the SEER cohort. On a cohort of 895 osteosarcoma patients, DeepOSSA achieves an AUC of 0.8058±0.0081 and an F1-score of 0.6506±0.0060 , exceeding seven representative baselines from classical machine learning, general-purpose deep learning, and dedicated survival analysis. We further benchmark DeepOSSA against two previously published externally validated Cox nomograms (Liu et al., 2023b; Yang et al., 2021), demonstrating competitive or superior performance across multiple survival-specific metrics. We also conduct ablation studies, statistical significance testing, and SHapley Additive exPlanations (SHAP)-based interpretability analysis to provide a comprehensive evaluation. We further show that the advantage is consistent across the entire follow-up window and translates into clinically meaningful individualized risk stratification.

## 2 Materials and methods

### 2.1 Data source and cohort selection

#### 2.1.1 Data retrieval and ethics

Data were retrieved from the Surveillance, Epidemiology, and End Results (SEER) database (https://seer.cancer.gov/) following an approved access request. We utilized SEER*Stat software (version 8.3.5) to extract comprehensive patient information, including demographic characteristics, clinicopathological features, and treatment modalities (surgery, radiotherapy, and chemotherapy).

### 2.1.2 Inclusion and exclusion criteria

The inclusion criteria were defined as follows:

1. Histologically confirmed primary malignant osteosarcoma based on the International Classification of Diseases for Oncology (ICD-O-3) codes: 9180–9187, 9192–9194, and 9200.
2. Osteosarcoma identified as the first and only primary malignancy.
3. Confirmed metastatic status (lung, bone, brain, and liver) based on imaging or pathological examination.
4. Complete clinical and follow-up data, including age at diagnosis, sex, marital status, race, primary tumor site, tumor size, stage, grade, metastatic sites, and records of surgical or adjuvant therapies (radiotherapy and chemotherapy).
5. Known cause of death and clear survival time post-diagnosis.

The exclusion criteria included the following:

1. Incomplete clinicopathological or survival information.
2. Unknown tumor size, stage, or race.
3. Patients lost to follow-up or those with missing/null data entries.

### 2.1.3 Study design and population

This retrospective cohort study initially screened patients diagnosed with osteosarcoma between 2010 and 2018. The year 2010 was selected as the starting point because the SEER database began incorporating detailed site-specific metastasis information at that time. A total of 895 patients met the inclusion criteria and were randomly assigned to either a training set (n = 627) or a validation set (n = 268) at a 7:3 ratio.

The demographic, clinicopathological, and treatment-related characteristics of the osteosarcoma patients from the SEER database are summarized in Table 1. To better describe the data, we present a summary of the missing/unknown data for categorical variables in the full cohort in Table 2. To ensure the scientific validity of the data partitioning, we compared all clinical indicators between the training set and the validation set. Statistical analysis revealed no significant differences across all clinical variables between the two cohorts (all P>0.05 ), demonstrating that the training and validation sets possess high baseline comparability. This balance confirms that the data grouping is rational and provides a robust foundation for model development and subsequent performance evaluation.

**TABLE 1:** Baseline characteristics of osteosarcoma patients in the training and validation cohorts.

Variable	Total (n = 895)	Training (n = 627)	Validation (n = 268)	*p*-value
Age (median, Q1, Q3)	25 (14.5, 55)	24 (15, 56)	25.5 (14, 53)	0.577
Sex, n (%)	​	​	​	0.636
Male	480 (54)	340 (54)	140 (52)	​
Female	415 (46)	287 (46)	128 (48)	​
Race, n (%)	​	​	​	0.110
Black	144 (16)	94 (15)	50 (19)	​
White	648 (72)	453 (72)	195 (73)	​
Other	103 (12)	80 (13)	23 (9)	​
Marital status, n (%)	​	​	​	0.911
Single	543 (61)	382 (61)	161 (60)	​
Married	252 (28)	174 (28)	78 (29)	​
Divorced/widowed/other	100 (11)	71 (11)	29 (11)	​
Tumor size, n (%)	​	​	​	0.304
≤ 38 mm	59 (7)	35 (6)	24 (9)	​
38–160 mm	414 (46)	291 (46)	123 (46)	​
> 160 mm	62 (7)	45 (7)	17 (6)	​
Unable to evaluate	360 (40)	256 (41)	104 (39)	​
Primary site, n (%)	​	​	​	0.876
Upper extremity	93 (10)	63 (10)	30 (11)	​
Lower extremity	496 (55)	349 (56)	147 (55)	​
Spine/mandible/other	306 (34)	215 (34)	91 (34)	​
Laterality, n (%)	​	​	​	0.275
Left	381 (43)	259 (41)	122 (46)	​
Right	360 (40)	263 (42)	97 (36)	​
Not a paired site/other	154 (17)	105 (17)	49 (18)	​
Grade, n (%)	​	​	​	0.592
Well differentiated	25 (3)	20 (3)	5 (2)	​
Moderately differentiated	56 (6)	38 (6)	18 (7)	​
Poorly differentiated	177 (20)	120 (19)	57 (21)	​
Undifferentiated	365 (41)	263 (42)	102 (38)	​
Unknown	272 (30)	186 (30)	86 (32)	​
Stage, n (%)	​	​	​	0.597
Stage I	139 (16)	94 (15)	45 (17)	​
Stage II	266 (30)	184 (29)	82 (31)	​
Stage III	16 (2)	14 (2)	2 (1)	​
Stage IV	106 (12)	75 (12)	31 (12)	​
Unknown	368 (41)	260 (41)	108 (40)	​
T stage, n (%)	​	​	​	0.577
T1	225 (25)	152 (24)	73 (27)	​
T2	279 (31)	201 (32)	78 (29)	​
T3	7 (1)	4 (1)	3 (1)	​
TX	384 (43)	270 (43)	114 (43)	​
N stage, n (%)	​	​	​	0.817
N0	553 (62)	390 (62)	163 (61)	​
N1	11 (1)	7 (1)	4 (1)	​
NX	331 (37)	230 (37)	101 (38)	​
M stage, n (%)	​	​	​	0.792
M0	478 (53)	333 (53)	145 (54)	​
M1	100 (11)	73 (12)	27 (10)	​
Unknown	317 (35)	221 (35)	96 (36)	​
Surgery, n (%)	​	​	​	0.869
No	302 (34)	210 (33)	92 (34)	​
Yes	593 (66)	417 (67)	176 (66)	​
Radiation, n (%)	​	​	​	0.068
No	790 (88)	562 (90)	228 (85)	​
Yes	105 (12)	65 (10)	40 (15)	​
Chemotherapy, n (%)	​	​	​	1.000
No	212 (24)	149 (24)	63 (24)	​
Yes	683 (76)	478 (76)	205 (76)	​
Bone metastasis, n (%)	​	​	​	0.210
No	858 (96)	605 (96)	253 (94)	​
Yes	37 (4)	22 (4)	15 (6)	​
Brain metastasis, n (%)	​	​	​	1.000
No	890 (99)	623 (99)	267 (100)	​
Yes	5 (1)	4 (1)	1 (0)	​
Liver metastasis, n (%)	​	​	​	0.587
No	891 (100)	625 (100)	266 (99)	​
Yes	4 (0)	2 (0)	2 (1)	​
Lung metastasis, n (%)	​	​	​	0.264
No	751 (84)	520 (83)	231 (86)	​
Yes	144 (16)	107 (17)	37 (14)	​
Vital status, n (%)	​	​	​	0.292
Alive	556 (62)	382 (61)	174 (65)	​
Dead	339 (38)	245 (39)	94 (35)	​
Survival time (median, Q1, Q3)	27 (11, 55)	26 (11, 52)	30 (12, 58)	0.116

**TABLE 2:** Summary of missing/unknown data for categorical variables in the full cohort (N=895) .

Variable	Observed	Observed (%)	Missing/unknown	Missing (%)
Tumor size	535	60	360 (unable to evaluate)	40
Grade	623	70	272 (unknown)	30
AJCC stage	527	59	368 (unknown)	41
T Stage	511	57	384 (TX)	43
N stage	564	63	331 (NX)	37
M Stage	578	65	317 (unknown)	35
Age, sex, race, marital status	895	100	0	0
Surgery, radiation, chemotherapy	895	100	0	0
Bone/brain/liver/lung metastasis	895	100	0	0

### 2.2 Variable processing and discretization

To enhance model interpretability and accommodate the common practice of using categorical covariates in survival analysis, continuous variables were transformed into categorical ones using X-tile software, a data-driven tool that identifies optimal cut-off points. To prevent information leakage, X-tile was applied exclusively to the training set to determine the optimal cut-off points, which were then applied to the validation set without modification. Within the framework of Kaplan–Meier survival analysis, X-tile systematically evaluates all potential thresholds and determines the optimal split that maximizes intergroup survival differences based on the minimum p-value approach (Camp et al., 2004). For instance, tumor size was categorized into four groups: ≤ 38 mm, 38–160 mm, > 160 mm, and “Unable to evaluate” (coded as 999). The “Unable to evaluate” category preserves information from cases with missing or non-measurable lesions due to insufficient imaging data. A comprehensive summary of missing data patterns for all categorical variables is provided in Supplementary Table S2.

All categorical covariates, including age group, primary tumor site, TNM stage, and metastatic status, were encoded using one-hot encoding prior to model inclusion to avoid implicit assumptions about ordinality or spacing between categories. Furthermore, given the well-established prognostic significance of metastases in specific organs, binary indicator variables (i.e., presence vs. absence) were constructed for lung, bone, brain, and liver metastases to precisely capture their individual impact on survival outcomes.

### 2.3 Model architecture

As illustrated in Figure 1, the proposed DeepOSSA framework comprises three primary modules: a feature embedding layer, a representation learning layer, and an output layer. The feature embedding layer projects each one-hot encoded clinical variable into a dense token representation. The representation learning layer consists of a stacked multi-layer Transformer tower and an auxiliary debiasing tower, designed to extract high-level patient representations through dense, all-pairs feature crossing and to mitigate distributional bias from the raw embeddings, respectively. Finally, the output layer fuses the two representation branches and feeds them into a Cox-style discrete-time survival prediction head. The model is formally described in the sections that follow.

**FIGURE 1:**
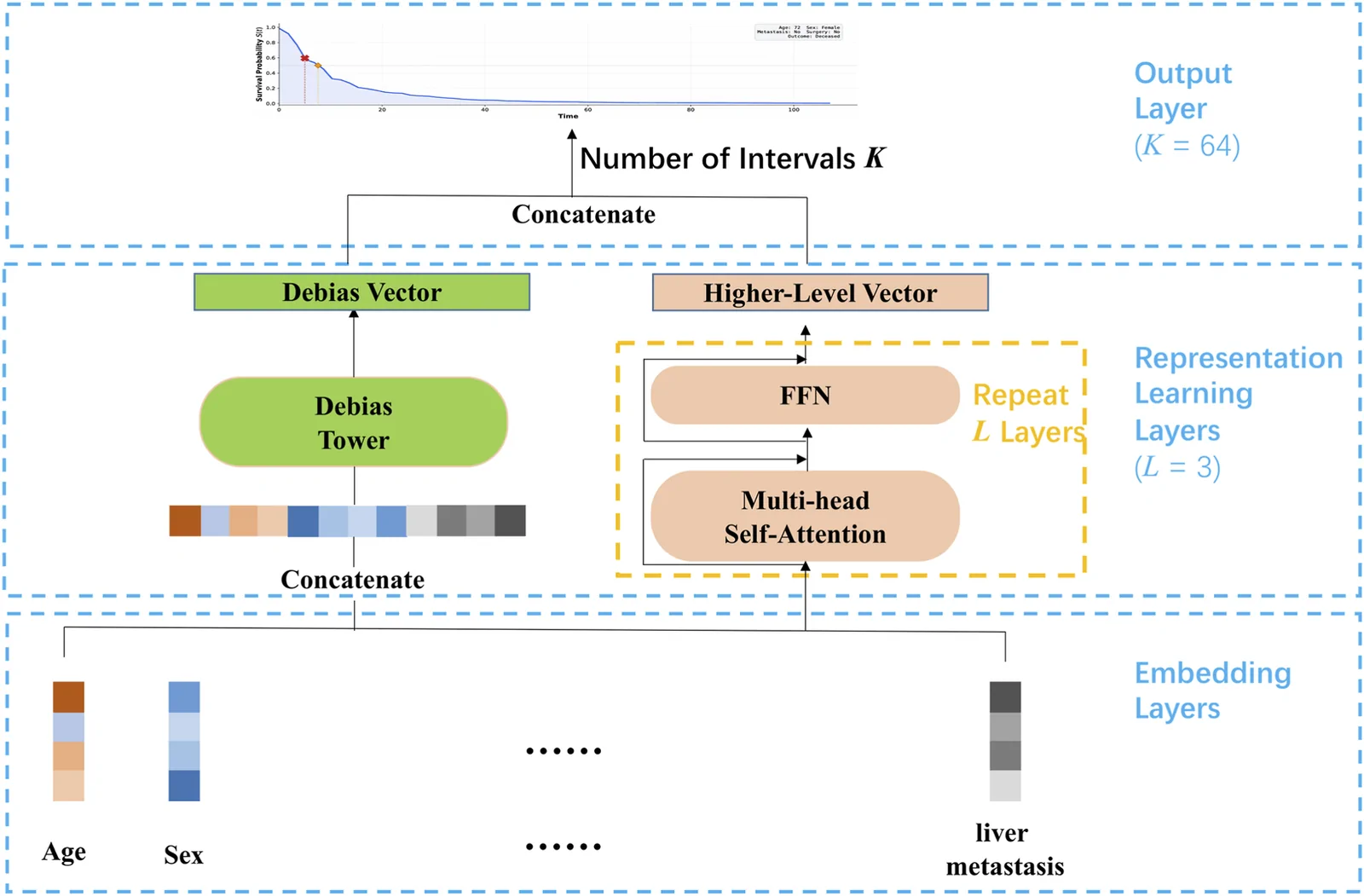
Overall architecture of the proposed DeepOSSA framework. One-hot encoded clinical variables are first projected into dense token embeddings by the feature embedding layer. The token sequence is then processed in parallel by a multi-layer Transformer-based representation tower, which performs dense, all-pairs crossing of sparse features via multi-head self-attention, and an auxiliary debiasing tower, which applies a single linear transformation to the raw embeddings to preserve first-order signals. The two branches are concatenated and fed into a Cox-style discrete-time survival prediction head, which is optimized end-to-end under a negative log-likelihood objective. Hyperparameter values shown in parentheses correspond to the configuration used in this study.

#### 2.3.1 Feature embedding layer

Given an input sample composed of D one-hot encoded clinical features, $x_{i}=\left(x_{1}^{i},x_{2}^{i},\ldots ,x_{D}^{i}\right)$ where each $x_{j}^{i}\in {\left\{0,1\right\}}^{N_{j}}$ is the one-hot encoding of feature j over its $N_{j}$ possible values, every feature is projected into a d -dimensional token representation via a feature-specific linear embedding as Equation 1:

$$e_{j}=W_{e,j}\;x_{j}^{i},$$ (1)

where $W_{e,j}\in R^{d\times N_{j}}$ is the learnable embedding matrix for feature j . This projection serves as the lookup-table-style embedding that converts each sparsely encoded clinical variable into a dense token suitable for downstream attention-based feature crossing.

#### 2.3.2 Transformer-based representation tower

The token sequence $E={\left(e_{1},e_{2},\ldots ,e_{D}\right)}^{⊤}\in R^{D\times d}$ produced by the feature embedding layer is first stabilized via batch normalization and augmented with sinusoidal positional encodings to preserve ordinal information among features (Chen Y et al., 2023; Kazemnejad et al., 2023) as Equation 2:

$$H^{\left(0\right)}=\text{PE}\;\left(\text{BN}\left(E\right)\right),$$ (2)

where BN(⋅) denotes batch normalization and PE(⋅) denotes sinusoidal positional encoding. The embedded sequence $H^{\left(0\right)}$ is fed into a multi-layer Transformer encoder to capture high-order feature interactions as Equation 3:

$$H^{\left(L\right)}={\text{Transformer}}_{L}\left(\ldots {\text{Transformer}}_{2}\left({\text{Transformer}}_{1}\left(H^{\left(0\right)}\right)\right)\ldots \right),$$ (3)

where $H^{\left(L\right)}\in R^{D\times d}$ represents the final output of the Transformer-based representation tower, ${\text{Transformer}}_{l}$ denotes the l th Transformer block, and L is a hyperparameter indicating the total number of stacked blocks. Each Transformer block is formally defined as Equations 4, 5:

$${\hat{H}}^{\left(l\right)}=LayerNorm\;\left(H^{\left(l-1\right)}+MHSA\;\left(H^{\left(l-1\right)}\right)\right),$$ (4)

$$H^{\left(l\right)}=LayerNorm\;\left({\hat{H}}^{\left(l\right)}+FFN\;\left({\hat{H}}^{\left(l\right)}\right)\right),$$ (5)

where LayerNorm(⋅) denotes the layer normalization function. MHSA(⋅) represents the multi-head self-attention mechanism configured with h parallel attention heads (Vaswani et al., 2017; Fan et al., 2024). The position-wise feedforward network (FFN) is defined as Equation 6:

$${FFN}^{\left(l\right)}\left({\hat{H}}^{\left(l\right)}\right)=W_{2}^{\left(l\right)}\;\sigma \left(W_{1}^{\left(l\right)}{\hat{H}}^{\left(l\right)}+b_{1}^{\left(l\right)}\right)+b_{2}^{\left(l\right)},$$ (6)

where $W_{2}^{\left(l\right)},W_{1}^{\left(l\right)}$ and $b_{2}^{\left(l\right)},b_{1}^{\left(l\right)}$ denote the learnable weight matrices and bias vectors of the FFN, respectively. Here, σ signifies the SiLU (Sigmoid Linear Unit) activation function. The additive terms in Equation 4 and Equation 5 constitute residual connections that preserve low-level clinical signals and stabilize gradient flow through the L -layer stack (He et al., 2016; Zeng et al., 2021). The final output $H\in R^{1\times Dd_{\text{model}}}$ is obtained by flattening and concatenating the high-level representations of individual features from the last Transformer layer $H^{\left(L\right)}\in R^{D\times d_{\text{model}}}$ , which yields a $D\cdot d_{\text{model}}$ -dimensional vector, formally expressed as Equation 7

$$H=Flatten\left(H^{\left(L\right)}\right).$$ (7)

#### 2.3.3 Debiasing tower

To mitigate potential feature biases in clinical data, we introduce an auxiliary debiasing tower that operates directly on the raw token embeddings E via a single linear transformation as Equation 8:

$$H_{\text{debias}}=W_{\text{debias}}\;\tilde{E}+b_{\text{debias}},$$ (8)

where $\tilde{E}=Concat\left(E\right)\in R^{1\times D\cdot d_{\text{model}}}$ denotes the concatenation of the individual feature embeddings into a unified token sequence and $W_{\text{debias}}\in R^{d_{\text{debias}}\times D\cdot d_{\text{model}}}$ and $b_{\text{debias}}\in R^{d_{\text{debias}}}$ are the learnable weight matrix and bias of the linear transformation, respectively. This shallow branch preserves first-order signals from the raw embeddings and provides a residual pathway that complements the deep, nonlinear representations produced by the Transformer tower.

#### 2.3.4 Output layer

The output layer first fuses the representations from the Transformer-based representation tower and the debiasing tower via concatenation as Equation 9:

$$H_{\text{fused}}=Concat\;\left(H,\;H_{\text{debias}}\right).$$ (9)

The fused representation is then passed through a shallow fully connected network to produce discrete-time conditional hazard estimates as Equation 10:

$$\hat{\phi }=W_{o}\;\sigma \;\left(W_{h}\;H_{\text{fused}}+b_{h}\right)+b_{o},$$ (10)

where $W_{h}$ and $W_{o}$ denote the learnable weight matrices. The output $\hat{\phi }$ represents the conditional logits for each time interval.

### 2.4 Model optimization

We follow a widely adopted setting and use negative log-likelihood (NLL) to optimize our survival neural networks (Katzman et al., 2018). Under the discrete-time framework, the model predicts a conditional hazard probability for each patient at each time interval k as Equation 11:

$$h_{i}\left(k\right)=sigmoid\left({\hat{\phi }}_{i,k}\right)=\frac{1}{1+\exp\left(-{\hat{\phi }}_{i,k}\right)},\;k=1,2,\ldots ,K,$$ (11)

where ${\hat{\phi }}_{i,k}$ is the k th logit value of the model output. From this, the cumulative distribution function (CDF) and survival function can be computed as Equation 12:

$$S_{i}\left(k\right)=\overset{k}{\underset{j=1}{\prod }}\left(1-h_{i}\left(j\right)\right),$$ (12)

where $S_{i}\left(k\right)$ represents the probability that patient i survives beyond time interval k . For a given observed sample $\left(x_{i},t_{i}^{*},\delta_{i}\right)$ (where $t_{i}^{*}$ is the censoring time index), the likelihood function is Equation 13:

$$\ell_{i}\left(\theta \right)=\left\{\begin{array}{cc} h_{i}\left(t_{i}^{*}\right)\overset{t_{i}^{*}-1}{\underset{k=1}{\prod }}\left(1-h_{i}\left(k\right)\right), & \text{if }\delta_{i}=1\left(\text{event observed}\right)\\ \overset{t_{i}^{*}}{\underset{k=1}{\prod }}\left(1-h_{i}\left(k\right)\right), & \text{if }\delta_{i}=0\left(\text{censored}\right) \end{array}\right..$$ (13)

Taking the logarithm and negating, we obtain the per-sample negative log-likelihood as Equation 14:

$$-\log\ell_{i}\left(\theta \right)=-\delta_{i}\log h_{i}\left(t_{i}^{*}\right)-\overset{t_{i}^{*}-\delta_{i}}{\underset{k=1}{\sum }}\log\left(1-h_{i}\left(k\right)\right).$$ (14)

The negative log-likelihood loss function over the entire training set is defined as Equation 15:

$$L_{\text{NLL}}\left(\theta \right)=-\frac{1}{N}\overset{N}{\underset{i=1}{\sum }}\left[\delta_{i}\log h_{i}\left(t_{i}^{*}\right)+\underset{k<t_{i}^{*}}{\sum }\log\left(1-h_{i}\left(k\right)\right)\right].$$ (15)

By employing Stochastic Gradient Descent (SGD) to optimize Equation 15, our model achieves high computational efficiency, enabling effective training on large-scale datasets (Amari, 1993; Fan et al., 2023).

### 2.5 Evaluation metrics

Our SEER osteosarcoma cohort exhibits a pronounced class imbalance: the number of surviving patients exceeds that of deceased patients by more than 60%. Under such conditions, conventional accuracy can be misleading, as it may reflect high performance simply by correctly predicting the majority class while failing to capture the model’s ability to identify the minority class.

To address this limitation, we adopt two robust evaluation metrics: the area under the curve (AUC) of the receiver operating characteristic and the F1-score. AUC measures the model’s ability to distinguish between classes across all classification thresholds, reflecting the area under the receiver operating characteristic (ROC) curve. It generally ranges from 0.5 (no discrimination) to 1.0 (perfect separation), with higher values indicating better discriminative power (Fan et al., 2022c; Chen J et al., 2023; Liu et al., 2024; Qian et al., 2023). The F1-score is the harmonic mean of precision and recall, providing a balanced measure that accounts for both false positives and false negatives. A higher F1-score also indicates better model performance (Fan et al., 2022a; Lin et al., 2026).

### 2.6 Baseline models

To rigorously evaluate the performance of DeepOSSA, we compare it against seven representative baseline methods spanning three methodological categories: classical machine learning, deep learning, and survival analysis.

#### 2.6.1 Classical machine learning models

- Support vector machine (SVM). SVM finds the maximum-margin hyperplane in a kernel-induced feature space to model nonlinear decision boundaries.
- Random forest (RF). RF aggregates predictions from an ensemble of independently trained decision trees via bootstrap aggregating, providing robust performance on heterogeneous tabular data.
- Gradient boosted decision trees (GBDT). GBDT sequentially builds an additive tree ensemble in which each new tree corrects the residual errors of the previous ones; we use XGBoost as its efficient implementation.

#### 2.6.2 Deep learning architectures

- Multi-layer perceptron (MLP). MLP is a feedforward network of stacked fully connected layers with nonlinear activations, capable of learning hierarchical feature representations from raw clinical variables.
- Transformer. The Transformer uses multi-head self-attention to capture global pairwise feature interactions, treating each clinical variable as an individual token for dynamic inter-feature relevance computation.

#### 2.6.3 Survival analysis models

- Cox proportional hazards model (CoxPH). CoxPH is a classical semi-parametric survival model that assumes a linear and multiplicative relationship between covariates and the hazard function.
- DeepSurv. DeepSurv extends CoxPH by replacing its linear predictor with a deep neural network, enabling nonlinear covariate modeling while retaining the proportional hazards framework.

By comparing DeepOSSA with these baselines, we aim to demonstrate its advantages in capturing complex nonlinear interactions over classical ML methods, its effectiveness of survival-specific design over general-purpose deep architectures, and its improvement over existing survival models through enhanced representation learning.

### 2.7 Implementation details

The proposed DeepOSSA framework is implemented in Python using PyTorch as the deep learning backend and the pycox library for discrete-time survival modeling (Paszke et al., 2019; Kvamme et al., 2019). All neural network modules, including the feature embedding layer, the Transformer encoder, the auxiliary debiasing tower, and the survival prediction head, are built upon PyTorch’s dynamic computational graph, which facilitates flexible model construction and efficient GPU-accelerated training. Feature preprocessing and standardization are performed with Scikit-Learn, which is also used to compute all evaluation metrics (AUC, F1 score, and C-index) (Pedregosa et al., 2011).

Hyperparameter optimization follows a grid search strategy. The key hyperparameters and their search ranges are as follows: embedding dimension $d_{\text{model}}\in \left\{12,16,20,24,28,32\right\}$ , number of attention heads $n_{\text{head}}\in \left\{1,2,4\right\}$ , learning rate $\eta \in \left\{0.05,0.02,0.01,0.005,0.001,5\times 10^{-4}\right\}$ , batch size B∈{32,64,128} , and dropout rate p∈{0.05,0.1,0.15,0.2,0.3} . All models are trained for a maximum of E=800 epochs with the Adam optimizer, and the best checkpoint is selected based on the validation loss monitored by an early stopping callback. The complete set of selected hyperparameters is listed in Supplementary Table S1.

## 3 Results

### 3.1 Overall performance

To improve the robustness and stability of our performance estimates, we evaluate all models using repeated random splits: at each run, the dataset is randomly partitioned into training and test sets at a 7:3 ratio, and the entire training–evaluation pipeline is repeated five times. Results are reported as mean ± standard deviation across the five runs. We report the AUC, C-index, F1 score, and their corresponding relative improvements in Table 3, along with the detailed ROC curves in Figure 2. Note that the relative improvement in AUC is computed following a widely adopted convention (Zhou et al., 2018; Fan et al., 2022b), which measures the gain relative to the baseline’s effective discriminative margin above random chance. DeepOSSA consistently outperforms all baseline methods across both evaluation metrics, achieving the highest AUC of 0.8058±0.0081 and F1 score of 0.6506±0.0060 . Specifically, the relative improvement in Δ AUC ranges from +3.84% (over DeepSurv) to +34.42% (over SVM), with an average gain of +13.11% across all baselines. Statistical significance testing via paired t-tests confirms that DeepOSSA’s improvements over all baselines are statistically significant, with P values ranging from $6.0\times 10^{-5}$ (vs. GBDT) to $6.1\times 10^{-3}$ (vs. Transformer) and Cohen’s d effect sizes from 2.65 to 8.83 (Supplementary Table S2). DeLong tests further corroborate these findings, with all pairwise comparisons reaching significance ( p<0.05 ; p -values from $6.3\times 10^{-6}$ vs. SVM to 0.045 vs. DeepSurv; Supplementary Table S3). Notably, DeepOSSA is among the models that surpass the 0.8 AUC threshold, which has been widely adopted as a benchmark for good discriminative ability in clinical prediction models (Steyerberg, 2019; Collins et al., 2015), demonstrating strong discriminative capability for osteosarcoma survival prediction.

**TABLE 3:** Performance comparison of all models on the SEER osteosarcoma dataset, evaluated via repeated random splits with a 7:3 train–test split over five runs (mean ± std). Statistical significance of DeepOSSA vs. each baseline is assessed via a paired t-test. The best results are highlighted in **bold**.

Category	Model	AUC	C-index	F1 score	Sig
Classical ML	SVM	0.7275±0.0057	0.7477±0.0024	0.5675±0.0127	***
	RF	0.7760±0.0032	0.7689±0.0023	0.5956±0.0110	**
	GBDT	0.7566±0.0089	0.7575±0.0078	0.5895±0.0091	***
Deep learning	MLP	0.7783±0.0064	0.7604±0.0035	0.5892±0.0082	***
	Transformer	0.7924±0.0027	0.7720±0.0052	0.6026±0.0072	**
Survival analysis	CoxPH	0.7803±0.0063	0.7594±0.0037	0.6191±0.0101	***
	DeepSurv	0.7945±0.0041	0.7826±0.0042	0.6185±0.0108	**
Published nomograms (reproduced)					
​	Liu et al. (2023b)	0.7945±0.0012	0.7870±0.0073	0.5891±0.0084	*
​	Yang et al. (2021)	0.7405±0.0047	0.7453±0.0060	0.5805±0.0139	***
**Ours**	**DeepOSSA**	0.8058±0.0081	0.7889±0.0069	0.6506±0.0060	—

Sig.: Paired t-test significance (*** p<0.001 , ** p<0.01 , * p<0.05 ).

All metrics were computed via repeated random splits (7:3 split, five runs).

**FIGURE 2:**
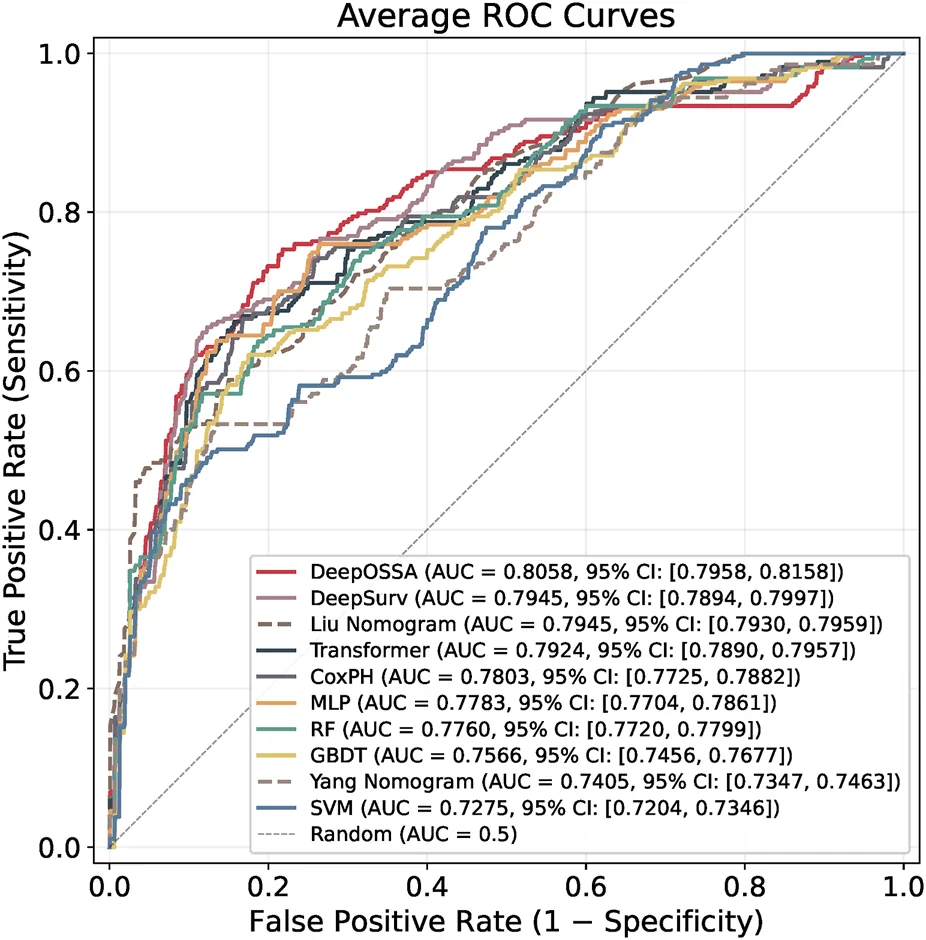
Average receiver operating characteristic (ROC) curves and corresponding AUC values with 95% confidence intervals for the proposed DeepOSSA model, seven baseline methods, and two published nomograms, computed across five repeated random splits. DeepOSSA achieves the highest AUC (exceeding 0.8) among all evaluated models.

We also compare DeepOSSA with two previously published Cox regression nomograms by Liu et al. (2023b) and Yang et al. (2021) by reproducing their models on our preprocessed cohort. As shown in Table 3, DeepOSSA outperforms both nomograms in AUC and F1 score. We note that the AUC values of the reproduced nomograms differ slightly from those reported in the original publications, which is attributable to differences in data preprocessing, exclusion criteria, and cohort composition after our filtering pipeline. Compared with these interpretable linear nomograms, DeepOSSA’s Transformer-based architecture captures high-order nonlinear feature interactions without relying on the proportional hazards assumption, while the auxiliary debiasing tower provides an additional mechanism to mitigate distributional biases inherent in registry data.

Compared with models that rely on linear assumptions or piecewise-constant decision boundaries, such as SVM ( Δ AUC = +34.42%), CoxPH ( Δ AUC = +9.10%), and GBDT ( Δ AUC = +19.17%), DeepOSSA exhibits substantial performance gains. This advantage stems from DeepOSSA’s deep nonlinear architecture, which captures complex, higher-order feature interactions that linear models and tree-based ensembles cannot adequately represent. In osteosarcoma prognosis, patient survival is influenced by intricate nonlinear relationships among clinical variables such as tumor stage, metastasis status, and treatment modality. The hierarchical representation learning in DeepOSSA effectively models these entangled dependencies, whereas SVM is constrained by its kernel-defined feature mapping and CoxPH is limited by its strict proportional hazards and linearity assumptions.

When compared against general-purpose deep learning models, DeepOSSA achieves a +9.88% Δ AUC improvement over the standard MLP and a +4.58% improvement over the Transformer. Although both MLP and Transformer possess strong nonlinear fitting capabilities, they lack survival-specific inductive biases. DeepOSSA addresses this by integrating the discrete-time logistic hazard formulation directly into its output layer, enabling the network to jointly learn feature representations and temporal survival dynamics under a unified Cox-style negative log-likelihood. Furthermore, the auxiliary debiasing tower acts as a complementary linear pathway that preserves clinically meaningful first-order feature signals alongside the deep nonlinear representations, preventing the deep tower from collapsing onto a narrow subspace that captures only mortality-associated patterns. This dual-branch design allows DeepOSSA to learn richer and more generalizable representations than architectures trained solely on classification or regression losses.

DeepOSSA also surpasses dedicated survival analysis methods, achieving Δ AUC improvements of +9.10% over CoxPH and +3.84% over DeepSurv. Although DeepSurv extends the Cox framework with a neural network predictor, it still operates under the proportional hazards assumption and relies on a single-branch architecture without explicit mechanisms for distributional bias correction. DeepOSSA overcomes these limitations through three key architectural innovations: (1) the Transformer-based encoder captures global all-pairs feature interactions via self-attention, dynamically adapting to patient-specific interaction patterns rather than relying on fixed-weight transformations; (2) the auxiliary debiasing tower independently learns bias correction signals from the raw token embeddings, mitigating the distributional imbalances inherent in clinical registries such as SEER; and (3) the joint optimization of the Transformer representation and a Cox-style discrete-time hazard head distills the most survival-relevant features into a compact latent code, enhancing both predictive accuracy and generalization on the limited sample size of 895 osteosarcoma patients.

### 3.2 Ablation study

To quantify the contribution of each architectural component, we conduct an ablation study evaluating five variants of DeepOSSA: (i) the full model, (ii) without the debias tower, (iii) without Transformer attention (replaced by standard MLP), (iv) with standard MLP embeddings (embedding and pooling), and (v) with an alternative survival head. All variants are trained from scratch with identical hyperparameters and evaluated via repeated random splits with a 7:3 train–test split over five runs.

As shown in Table 4, removing the debias tower leads to a noticeable performance drop (AUC decreases from 0.806 to 0.770, F1 from 0.651 to 0.614) with substantially higher variance, confirming that the auxiliary linear pathway is important for preserving first-order signals and stabilizing training. Replacing the Transformer-based attention with a standard MLP also results in lower AUC (0.746 vs. 0.806) and higher variance, suggesting that self-attention provides more stable and effective feature interactions. The alternative survival head variant shows a slightly lower AUC (0.743), indicating that the residual connection in the output layer contributes to discriminative performance. Overall, the full DeepOSSA achieves the highest AUC and F1 among all variants, while maintaining a competitive C-index, confirming that the integration of all components yields the strongest discriminative and classification performance.

**TABLE 4:** Ablation study of the proposed DeepOSSA framework. All models are trained from scratch with identical hyperparameters and evaluated via repeated random splits with a 7:3 train–test split over five runs (mean ± std).

Variant	AUC	F1	C-index
Full DeepOSSA	0.806±0.0081	0.651±0.0060	0.789±0.0069
W/o debias tower	0.770±0.089	0.614±0.087	0.784±0.021
W/o Transformer attention (MLP)	0.746±0.041	0.586±0.055	0.782±0.010
Standard MLP embedding and pooling	0.758±0.031	0.602±0.027	0.781±0.012
Alternative survival head	0.743±0.014	0.582±0.027	0.786±0.011

### 3.3 SHAP-based interpretability analysis

To elucidate the decision-making mechanism of DeepOSSA and quantify the contribution of each clinical covariate to individual survival risk predictions, we applied SHapley Additive exPlanations (SHAP) analysis using the KernelExplainer framework with 50 randomly sampled background patients from the training cohort. For each of the 268 test patients, the SHAP value of a feature measures the marginal contribution of that feature to the predicted risk score relative to the population baseline.

The mean absolute SHAP values across all test samples reveal a clear hierarchy of prognostic determinants: age, pulmonary metastasis, and surgical intervention emerge as the three most influential features. This hierarchy is broadly consistent with prognostic variables selected in recent machine learning and nomogram-based studies on osteosarcoma survival (Spreafico et al., 2024b; Yang et al., 2021).

(i) Age as the dominant prognostic driver. Age emerged as the single most influential feature (mean |SHAP|=0.892 ), exhibiting a monotonic positive association with predicted risk (Figure 3). Patients with standardized age values exceeding 1.0 (corresponding to approximately ≥60 years) consistently received SHAP values above +0.8 , indicating strong positive risk attribution. This finding is consistent with the broader oncological literature recognizing advanced age as an adverse prognostic factor in older osteosarcoma patients, who exhibit diminished immune surveillance, reduced tolerance to intensive chemotherapy regimens, and a higher prevalence of comorbidities (Yang et al., 2021; Liu et al., 2023b). Notably, Spreafico et al. (2024b) found that age did not reach significance in the EURAMOS-1 cohort (restricted to patients aged ≤40 ), suggesting that the prognostic impact of age is most pronounced when the full age spectrum is represented, as in the SEER cohort used here.

**FIGURE 3:**
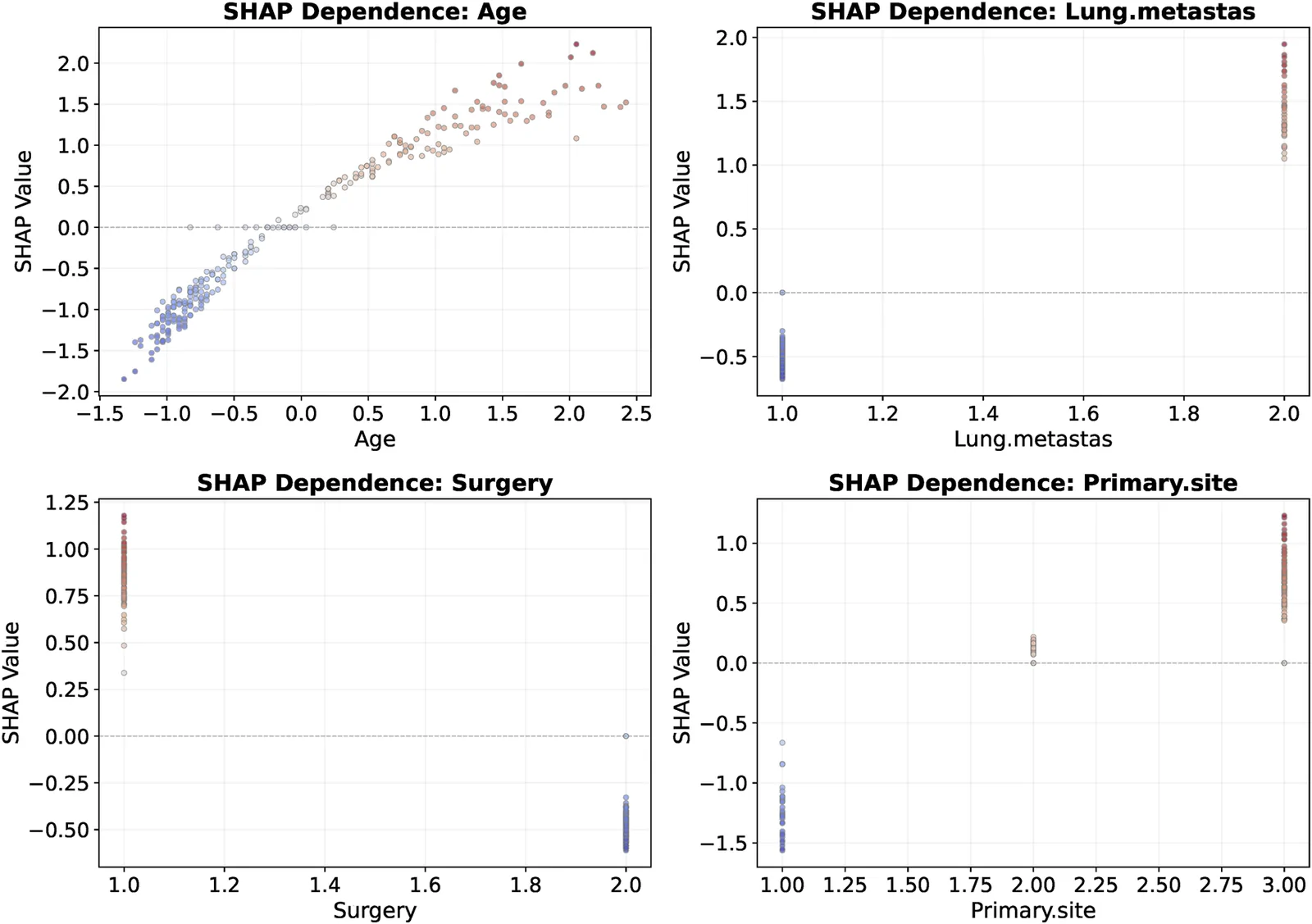
SHAP dependence plots for the top-4 features, showing the directional impact of each clinical variable on predicted risk.

(ii) Pulmonary metastasis as a critical risk amplifier. Lung metastasis ranked second in importance (mean |SHAP|=0.625 ), with affected patients receiving a mean SHAP of +1.45 , which is the highest positive attribution among all features (Figure 3). The lung represents the most common site of hematogenous dissemination in osteosarcoma, and pulmonary metastasis at diagnosis is widely recognized as a powerful adverse prognostic factor. In the EURAMOS-1 trial, the presence of lung metastases yielded the highest hazard ratio among all covariates (HR = 2.42, 95% CI: 1.95–3.02) (Spreafico et al., 2024b), and multiple SEER-based nomograms have identified metastatic status as a leading predictor of cancer-specific survival (Liu et al., 2023b; Yang et al., 2021).

(iii) Surgical intervention as a protective factor. Surgery ranked third (mean |SHAP|=0.612 ), functioning as a protective feature: patients who underwent surgical resection exhibited a mean SHAP of −0.48 (reduced risk), while those without surgery received +0.86 (elevated risk; Figure 3). This is consistent with the central role of surgical resection in osteosarcoma management: complete surgical removal of the primary tumor is a prerequisite for curative intent. In the EURAMOS-1 cohort, intralesional or unknown-margin surgical excision was associated with elevated hazard (HR = 1.43, 95% CI: 1.00–2.05) relative to wide/radical resection (Spreafico et al., 2024b), and both the Liu et al. (2023b) and Yang et al. (2021) nomograms include surgical treatment as a significant predictor.

Collectively, the SHAP analysis confirms that DeepOSSA’s risk predictions are driven by clinically established prognostic factors, including age, pulmonary metastasis, surgical treatment, and tumor staging, rather than spurious or artifactual features. The directional consistency between SHAP attributions and known clinical associations (Figure 4) provides strong evidence that the Transformer-based architecture has learned interpretable, medically grounded decision boundaries, supporting the potential translatability of DeepOSSA as a transparent clinical decision-support tool.

**FIGURE 4:**
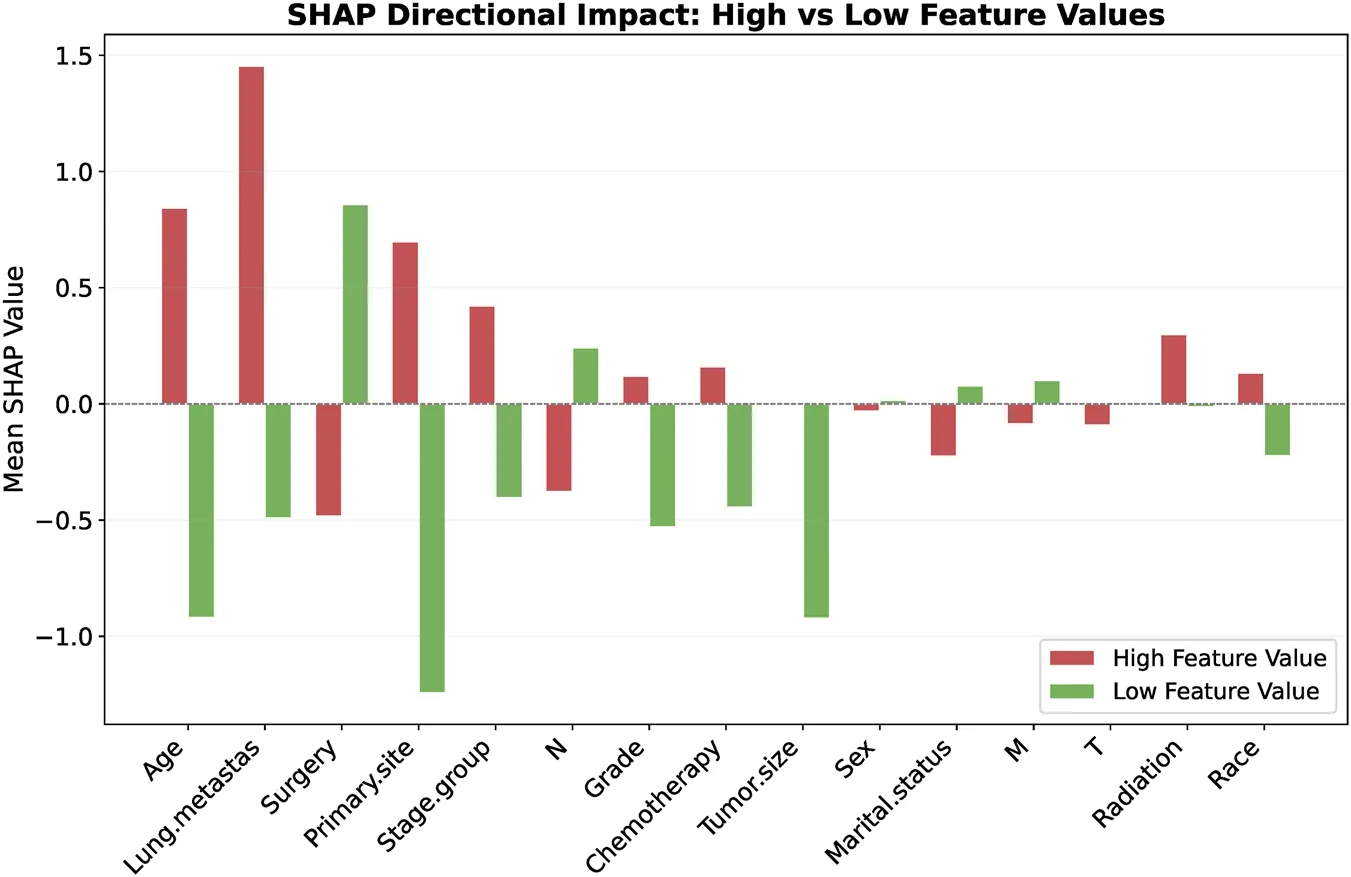
Directional SHAP impact: mean SHAP values for high vs. low feature values, demonstrating that the model correctly captures clinically established risk directions.

### 3.4 Performance with respect to survival time

To evaluate the temporal robustness of DeepOSSA’s predictive capability, we compute the area under the ROC curve (AUC) and F1 score at each discrete survival time point and compare the time-resolved trajectories of DeepOSSA against the CoxPH. Predictions are generated from the held-out test set of the SEER osteosarcoma cohort.

The AUC trajectory reveals a clear and persistent advantage of DeepOSSA over CoxPH at virtually every time point. DeepOSSA attains an overall mean AUC of 0.8076 (±0.0108) , compared with 0.7818 (±0.0060) for CoxPH.

Even within the first 20 months, when event density is typically highest in osteosarcomas, DeepOSSA achieves an AUC of 0.7927, exceeding CoxPH by +0.0172 . This early margin is clinically consequential: accurate risk stratification shortly after diagnosis directly informs adjuvant therapy decisions. Only at a single time anchor ( t=1.9 months) does CoxPH register a negligible and transient AUC advantage of −0.0017 , which is well within the noise floor of both models.

In the F1 score trajectory, DeepOSSA exhibits a mean deficit of −0.0284 relative to CoxPH within this same early window ( t≤20 months). We acknowledge that this F1 deficit is larger in absolute magnitude than the early AUC advantage of +0.0172 highlighted above, and both metrics should be interpreted with equal rigor. The early F1 deficit likely reflects the combination of (i) the relatively small number of survival events in this early window, which amplifies metric variance, and (ii) DeepOSSA’s joint optimization of representation learning and discrete-time hazard estimation, which during initial training epochs may trade off a small amount of classification sharpness for better-calibrated long-term survival probability estimates. Importantly, this early F1 lag is transient and reverses by the 20-month mark, after which DeepOSSA never trails CoxPH in F1 for any subsequent time anchor.

The AUC gap widens progressively: +0.0274 in the mid-term (20–60 months) and +0.0284 in the long-term ( >60 months). This monotonic expansion indicates that DeepOSSA’s learned representations not only generalize across time but actively benefit from the accumulation of temporal information. The maximum per-point AUC gain reaches +0.0343 at t=93.6 months, highlighting that DeepOSSA’s advantage is most pronounced precisely where CoxPH’s linear proportional-hazards assumption is most strained: in the long-tail survival regime.

The sustained AUC lead originates from DeepOSSA’s coupling of a Transformer-based representation tower with a Cox-style discrete-time hazard head, which jointly optimizes high-order feature crossing and time-resolved hazard prediction. Unlike CoxPH, which imposes a rigid log-linear relationship between covariates and hazard, DeepOSSA learns nonlinear, time-aware interaction patterns through attention-based representations of one-hot encoded clinical variables. This enables the model to capture complex temporal dynamics, such as time-varying treatment effects and competing-risk-like behaviors, that linear survival models cannot express.

The F1 score trajectory complements the AUC picture. Over the full timeline, DeepOSSA achieves a mean F1 of 0.5871 versus 0.5676 for CoxPH (+0.0195) and leads in 28 of 31 anchors (90.3%) . From the mid-term onward (20–100), DeepOSSA holds a consistent F1 advantage of +0.027 – +0.035 , mirroring the AUC trend.

As discussed above, the early-phase F1 deficit ( t≤20 months) is transient and attributable to event scarcity and the model’s emphasis on long-term calibration. After the 20-month mark, DeepOSSA consistently outperforms CoxPH in F1, with the advantage reaching statistical and clinical significance in the mid-to-late phase.

### 3.5 Visualization

To evaluate whether DeepOSSA learns semantically organized high-level representations, we projected the fused patient embeddings $H_{\text{fused}}$ (extracted before the survival prediction head) onto a 2D manifold using t-SNE and assessed the alignment between learned embedding geometry and survival outcomes (Van der Maaten and Hinton, 2008; Fan et al., 2022b).

Two key observations support the efficiency of the learned representations:

(i) Cluster separation by outcome. The died (n=94) and censored/alive (n=174) groups form spatially distinguishable clusters in the t-SNE projection. Patients who died (red) concentrate in regions of high predicted risk, while censored/surviving patients (green) occupy lower-risk regions. The substantial centroid distance (d=14.83) indicates that the Transformer encoder has actively learned features carrying survival-discriminative information, rather than encoding non-specific noise.

(ii) Continuous risk gradient. When colored by the model’s continuous predicted risk score (Figure 5b), patients transition smoothly from low-risk (blue, risk≲0.3 ) to high-risk (red, risk≳0.7 ) regions, forming a monotonic spatial gradient. The strong concordance between ground-truth outcome (subfigure A) and predicted risk (subfigure B) validates the entire end-to-end learning pipeline. The Transformer encoder produces representations, and the survival prediction head correctly maps those representations to prognostically meaningful risk scores.

**FIGURE 5:**
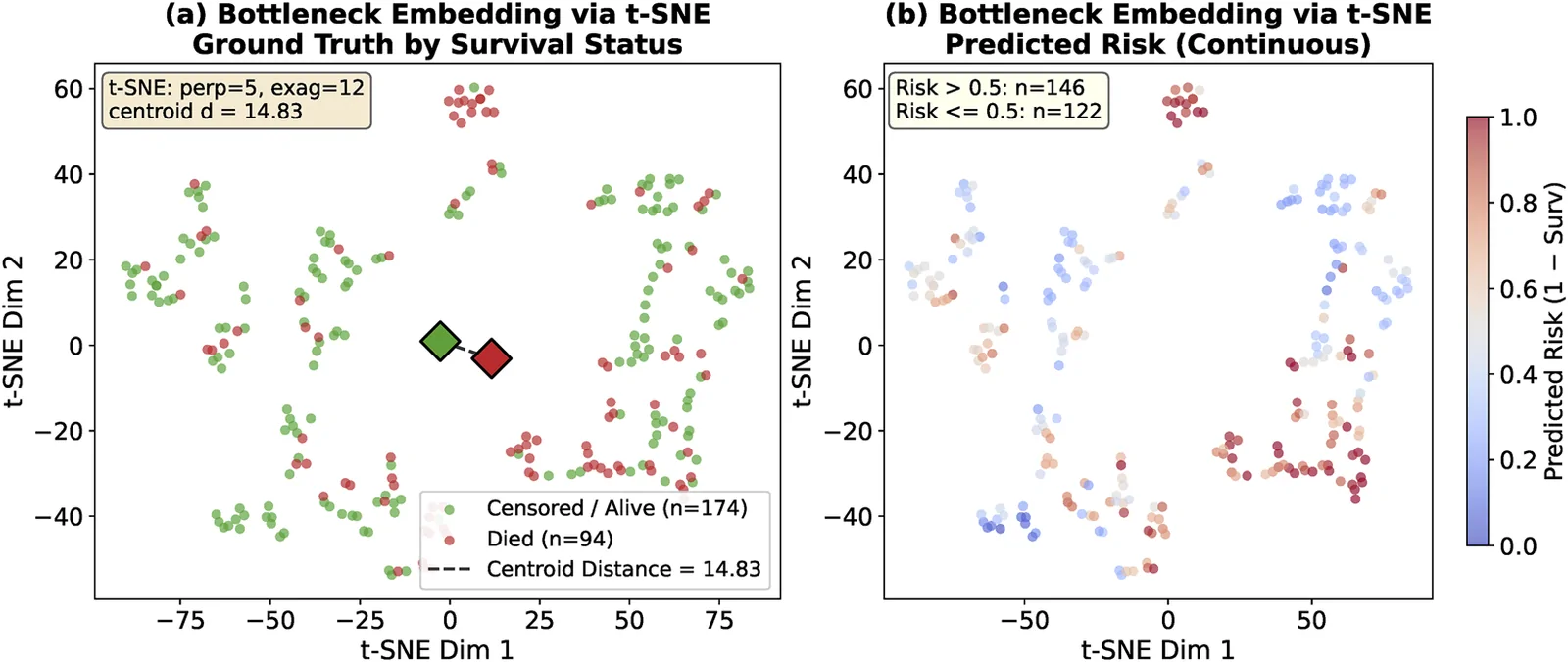
t-SNE visualization of the fused patient embeddings $H_{\text{fused}}$ learned by DeepOSSA on the held-out SEER osteosarcoma test set (n=268) . **(a)** Patients colored by ground-truth outcome (red: died, n=94 ; green: alive/censored, n=174 ); the two outcome groups form spatially distinguishable clusters with a centroid distance of d=14.83 . **(b)** The same projection colored by DeepOSSA’s continuous predicted risk score, showing a smooth, monotonic gradient from low-risk (blue) to high-risk (red) regions concordant with the ground-truth outcome distribution in subfigure **(a)**.

These results demonstrate that self-attention-based crossing of sparsely encoded clinical variables, coupled with a Cox-style discrete-time hazard head, enables DeepOSSA to capture nonlinear feature interactions (e.g., the prognostic significance of distant metastasis modulated by surgery and chemotherapy status) that linear Cox models cannot represent. The fused embedding constructs a multi-dimensional manifold in which patients with similar Cox risk scores but distinct clinical feature compositions occupy different regions, providing a richer representational basis for personalized survival prediction in osteosarcoma.

### 3.6 Case study: individualized risk stratification

To further illustrate the clinical interpretability of DeepOSSA, we present a representative case study comparing two patients from the held-out test set (N=268) with divergent survival trajectories. Their clinical profiles and model predictions are summarized in Table 5.

**TABLE 5:** Clinical profiles and DeepOSSA predictions for two representative patients.

Characteristic	Patient A	Patient B
Age/sex	13/male	17/female
Grade	IV	IV
Stage	IV	IV
Distant metastasis	Yes (bone, lung, liver)	Yes
Surgery	No	Yes
Chemotherapy	No	Yes
Predicted risk	0.970 (high)	0.460 (low)
Survival probability	0.030	0.540
Observed outcome	Died at 10 months	Alive at 107 months (censored)

Patient A was a 13-year-old male diagnosed with Grade IV osteosarcoma (T4N1M1) with multi-site metastasis involving bone, lung, and liver, who received neither surgical resection nor chemotherapy. DeepOSSA assigned a predicted risk of 0.970, corresponding to a final survival probability of only 0.030. The patient died at 10 months, consistent with the aggressive clinical presentation. As shown in Figure 6a, the predicted survival curve exhibits a steep, monotonic decline that drops below 0.5 within the first time interval, concordant with the patient’s advanced stage and distant metastasis, which are two well-established adverse prognostic factors in osteosarcoma.

**FIGURE 6:**
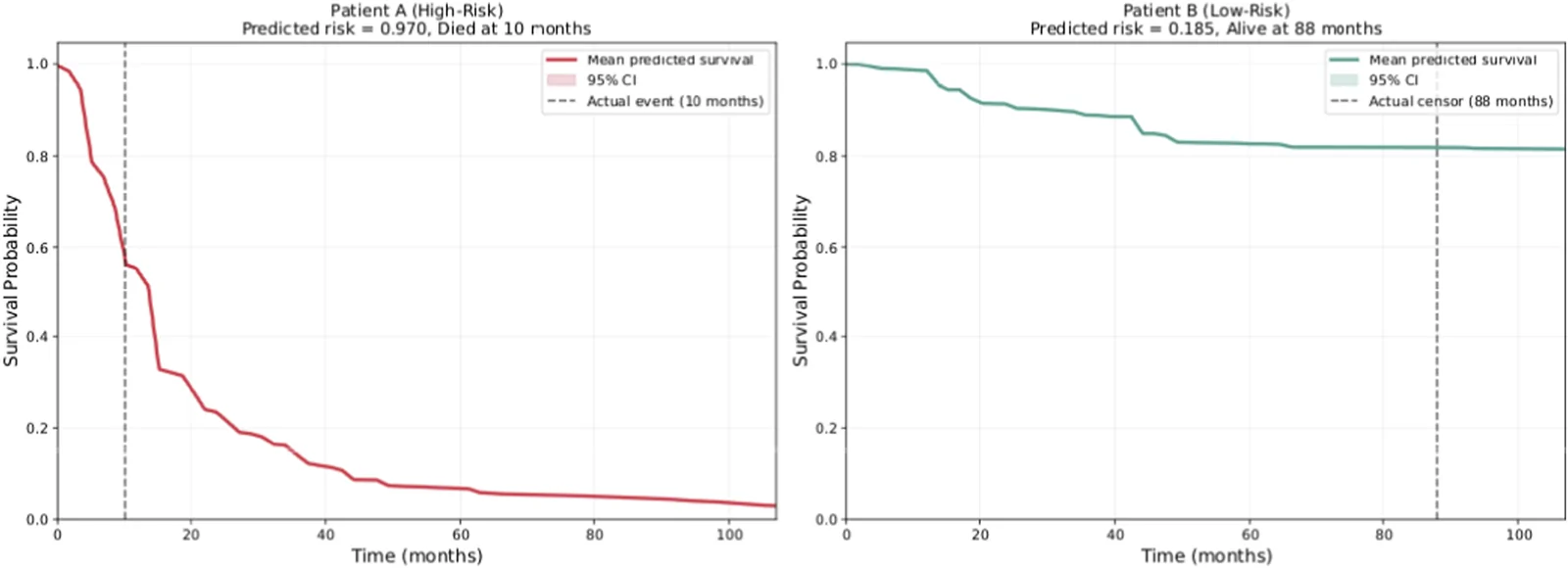
DeepOSSA-predicted survival curves for the two representative osteosarcoma patients summarized in Table 5. **(a)** Patient A (high-risk; predicted risk = 0.970, died at 10 months): the predicted survival probability declines steeply within the first time interval, consistent with the patient’s untreated multi-site metastatic disease. **(b)** Patient B (low-risk; predicted risk = 0.460, alive at 107 months and censored): the predicted survival curve declines gradually and plateaus near 0.5, reflecting the moderating effect of surgery and chemotherapy despite a clinically aggressive presentation.

Patient B, a 17-year-old female, presented with a comparably aggressive clinical profile (Grade IV, Stage IV, with documented metastasis). Despite this seemingly high-risk presentation, the patient survived at least 107 months and remained alive at last follow-up (censored). DeepOSSA assigned a substantially lower predicted risk of 0.460, with a survival probability of 0.540, correctly classifying this patient as low risk. Critically, Patient B received both surgery and chemotherapy, suggesting that treatment response mitigated the inherent clinical risk. The corresponding survival curve (Figure 6b) shows a gradual, modest decline that plateaus near 0.5, reflecting the model’s calibrated uncertainty for this borderline case.

The risk gap between these two patients (Δrisk=0.510) is substantial and clinically meaningful. More importantly, the model’s risk estimates align with the observed outcomes, which is a critical validation of its prognostic utility. This case study highlights two key strengths of the proposed framework:

1. Continuous risk calibration. Rather than producing a binary high/low classification, DeepOSSA outputs a continuous risk score (0.460 vs. 0.970), enabling clinicians to assess each patient on a risk spectrum and tailor follow-up intensity and treatment aggressiveness accordingly.
2. Feature-interaction awareness. Patient B’s low-risk assignment, despite Grade IV staging and distant metastasis, indicates that the Transformer encoder has learned to capture nonlinear feature interactions (e.g., the interplay among surgical intervention, chemotherapy response, and tumor biology) that traditional Cox regression cannot model. This ability to learn complex covariate interactions distinguishes DeepOSSA from conventional survival models and underscores its potential as an AI-assisted decision-support tool for personalized osteosarcoma management.

## 4 Discussion

### 4.1 Summary of key findings

In this study, we proposed DeepOSSA, a deep learning framework tailored for personalized survival prediction in osteosarcoma patients. The model is built on a cohort of 895 patients from the SEER database. It integrates a feature embedding layer, a Transformer-based representation tower, an auxiliary debiasing module, and a discrete-time Cox-style survival head. Rather than treating one-hot encoded features independently, our architecture uses multi-head self-attention to enable dense, all-pair interactions across sparse clinical variables. Comprehensive experiments against several state-of-the-art baselines spanning classical machine learning, deep learning, and dedicated survival analysis methods show that DeepOSSA achieves the highest AUC (0.8058±0.0081) and F1 score (0.6506±0.0060) among all models evaluated in this study. Time-dependent analyses further confirm that the predictive advantage of DeepOSSA is consistent across the entire follow-up window, while t-SNE visualization and a representative case study demonstrate that the learned representations carry semantically meaningful information. Ablation studies confirm the necessity of each architectural component, SHAP analysis reveals that the model’s predictions are driven by clinically established prognostic factors, and statistical significance testing via paired t-tests and DeLong tests validates the robustness of the reported improvements.

### 4.2 Comparison with published nomograms

Several externally validated Cox regression nomograms developed on SEER osteosarcoma data have achieved strong discriminative performance: Liu et al. (2023b) reported a training C-index of 0.80 with 3-/5-year AUC of 0.82/0.81, and Yang et al. (2021) achieved a development-cohort C-index of 0.818 with external validation C-index up to 0.843. These interpretable, linear models demonstrate that conventional approaches, when carefully designed and externally validated, can reach AUC levels comparable to those reported here. However, such models remain constrained by the proportional hazards assumption and cannot capture the high-order interactions among sparsely encoded categorical variables that DeepOSSA explicitly models via self-attention (Katzman et al., 2018; Liu et al., 2023a).

The empirical results suggest that DeepOSSA’s architectural design, coupling a Transformer-based representation tower with a Cox-style discrete-time hazard head and an auxiliary debiasing pathway, translates into tangible improvements in both discrimination and temporal robustness (Table 3; Figure 7). Crucially, the SHAP analysis (Section 3.3) reveals that the model’s risk predictions are anchored on clinically established prognostic factors, including age, pulmonary metastasis, and surgical intervention, rather than spurious features. The monotonic positive association between age and predicted risk, the strong positive attribution of lung metastasis, and the protective effect of surgery all align with decades of oncological evidence, providing confidence that the Transformer encoder has learned medically grounded decision boundaries. This interpretability is further reinforced by the t-SNE visualization (Section 3.5), which shows that the learned embedding space organizes patients along a continuous risk gradient concordant with ground-truth outcomes.

**FIGURE 7:**
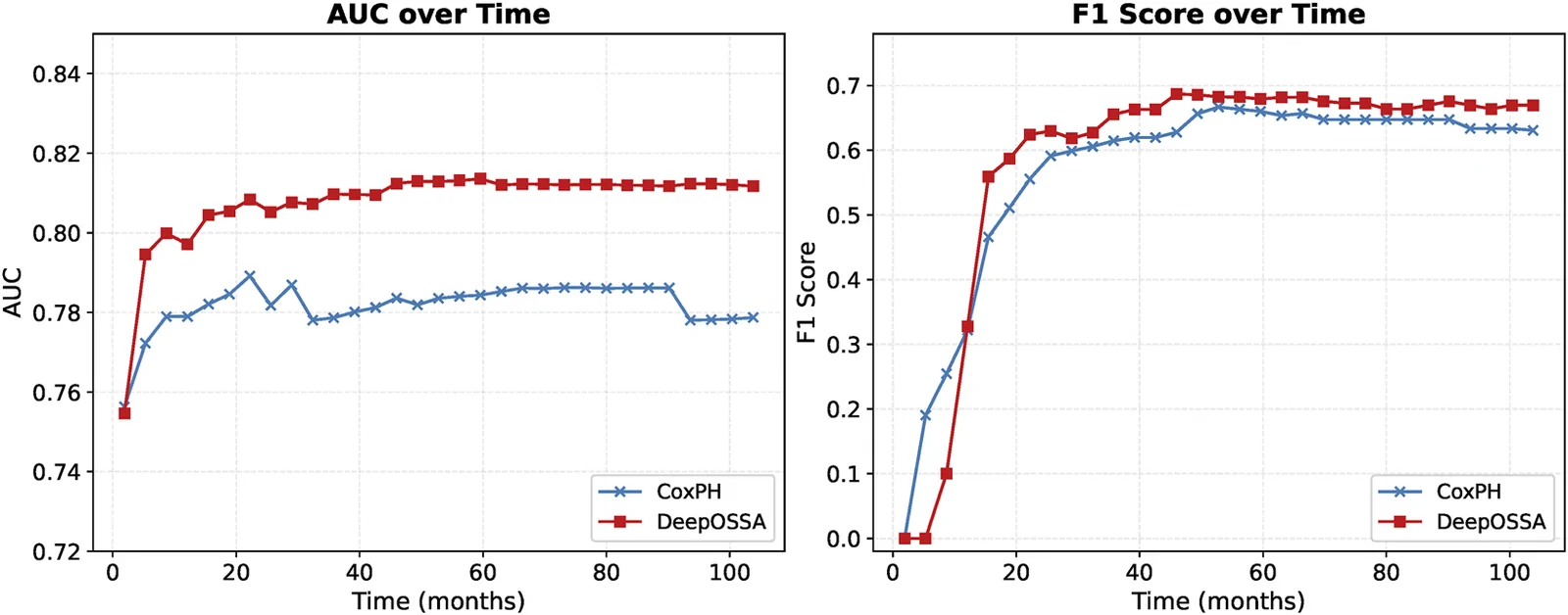
Time-dependent performance of CoxPH and DeepOSSA across the follow-up period on the held-out SEER osteosarcoma test set. Left: Time-dependent AUC trajectory; Right: time-dependent F1 score trajectory. DeepOSSA maintains a consistent AUC advantage at virtually every time anchor and a sustained F1 advantage from approximately 20 months onward.

While DeepOSSA performs competitively compared with published nomograms, an important distinction should be noted: the nomograms of Liu et al. (2023b) and Yang et al. (2021) were validated on independent external cohorts, whereas DeepOSSA was evaluated only on an internal 70:30 split. Direct comparison of AUC values across different validation schemes should therefore be interpreted cautiously. Future work should prioritize external validation of DeepOSSA on independent institutional or geographic cohorts to establish generalizability.

### 4.3 Clinical implications and generalizability

From a clinical perspective, DeepOSSA produces a continuous, well-calibrated risk score for each patient rather than a binary or coarse stage-based label. The case study contrasting Patient A and Patient B illustrates that the model can distinguish between two patients who appear similar under conventional staging yet exhibit markedly different outcomes due to differences in treatment exposure and feature interactions. This capability supports more nuanced individualized risk stratification, can inform decisions about the intensity of adjuvant therapy and the frequency of follow-up imaging, and may help identify high-risk patients who could benefit from earlier escalation of care or enrollment in clinical trials. The time-dependent evaluation further suggests that the model is suitable for longitudinal monitoring, where its advantage over CoxPH widens in the long-tail survival regime in which linear assumptions are most strained.

Although DeepOSSA was initially developed for osteosarcoma, its core design, particularly the ability to model high-order interactions among sparse categorical inputs, along with the auxiliary debiasing tower, is generalizable and can be adapted to other domains facing similar data constraints. For instance, researchers in cell and developmental biology often encounter comparable challenges, including mixed categorical covariates, heavily censored outcomes, and significant class imbalance (Zhang et al., 2020; Chen et al., 2021; Huang et al., 2025). In this context, DeepOSSA provides a reproducible methodological framework for survival modeling on complex, sparse clinical data. Moreover, the architecture offers a principled foundation for integrating registry-scale clinical variables with future multi-modal data sources such as radiology images, histopathology slides, and molecular profiles. This integration paves the way for further methodological innovation at the intersection of representation learning and survival analysis (Liu et al., 2024; Kong et al., 2025; Saraf et al., 2025; Lin et al., 2026).

### 4.4 Limitations

Several limitations should be acknowledged when interpreting the present results:

1. External validation. Although 895 patients constitute a sizeable cohort by osteosarcoma standards, this sample size remains modest for deep neural networks. Osteosarcoma is a rare disease, and publicly available large-scale cohorts are limited in number and geographic diversity, making external validation particularly challenging. Although we report performance over multiple repeated random splits (five runs with a 7:3 train–test split) to improve estimate stability, internal resampling does not fully demonstrate model generalizability, and the risk of overfitting to cohort-specific patterns cannot be fully excluded. External validation on independent cohorts (e.g., NCDB, institutional databases, or international registries) remains a critical next step.
2. Multi-modal extension. The current model uses only tabular clinical data. Future extensions should incorporate multi-modal data sources (e.g., radiology images, histopathology slides, and molecular profiles) to improve model performance and generalizability.

In future work, we plan to leverage transfer learning and domain adaptation techniques to mitigate these limitations and improve model generalization. Additionally, integrating world knowledge from pretrained large language models is expected to enhance zero-shot capabilities and interpretability (Ding et al., 2025).

In summary, we have introduced DeepOSSA in this work, a Transformer-based deep survival framework that performs dense crossing of sparsely encoded clinical variables and couples the resulting high-level representations with a Cox-style discrete-time hazard head for personalized survival prediction in osteosarcoma. On the SEER cohort, DeepOSSA achieves consistent and substantial improvements over classical machine learning, general-purpose deep learning, and dedicated survival analysis baselines, with particularly strong performance in long-term risk estimation and individualized risk stratification. While further validation on multi-institutional and multi-modal cohorts is needed before clinical deployment, the present results establish DeepOSSA as a promising step toward AI-assisted precision oncology for osteosarcoma and provide a transferable methodological foundation for survival modeling in other rare and heterogeneous malignancies.

## Data Availability

The original contributions presented in the study are included in the article/Supplementary Material; further inquiries can be directed to the corresponding author.
