# Supplementary material for: DeepOSSA: a deep learning framework for personalized survival prediction in osteosarcoma patients
Source: Front Cell Dev Biol. 2026 Oct 22;14:1868333. doi: 10.3389/fcell.2026.1868333 (PMC13735625; doi:10.3389/fcell.2026.1868333)
Supplement: Supplementary file 1 [file DataSheet1.pdf]

Supplementary Material

DeepOSSA — A Deep Learning Framework for Osteosarcoma Survival Prediction

Table S1: Hyperparameters of the proposed DeepOSSA-Transformer model.

**Category**	**Hyperparameter**	**Symbol**	**Value**
**Architecture**	Embedding dimension	d_{model}	24
	Attention heads	n_{head}	2
	Encoder layers	L	3
	Feed-forward dimension	d_{ff}	64
	Dropout rate	p	0.1
**Training**	Learning rate	η	0.015
	Batch size	B	32
	Max epochs	E	800
	Early stopping patience	P	12
**Components**	Optimizer	–	Adam
	Transformer activation	–	GELU
	Positional encoding	–	Sinusoidal
	Weight initialization	–	Xavier Uniform

Table S2: Statistical significance: DeepOSSA vs. baselines (paired t-test, 5 runs).

Comparison	ΔAUC	t-stat	t-test p	Cohen's d
vs SVM	+0.0783	14.024	0.000150 ***	7.012
vs RF	+0.0299	8.320	0.001140 **	4.160
vs GBDT	+0.0492	17.668	0.000060 ***	8.834
vs MLP	+0.0276	12.377	0.000245 ***	6.189
vs Transformer	+0.0135	5.292	0.006119 **	2.646
vs CoxPH	+0.0255	10.223	0.000516 ***	5.111
vs DeepSurv	+0.0113	5.927	0.004061 **	2.963

Table S3: DeLong test: DeepOSSA vs. baselines (overall risk prediction, AUC of DeepOSSA = 0.8116). Results are reported for a single representative test set, as the DeLong test operates on one ROC curve at a time.

Model	AUC (Baseline)	ΔAUC	p-value	Sig
SVM	0.7267	+0.0849	6.34×10^{-6}	***
RF	0.7699	+0.0417	0.0013	**
GBDT	0.7726	+0.0390	0.0248	*
MLP	0.7653	+0.0462	0.0069	**
Transformer	0.7732	+0.0384	0.0039	**
CoxPH	0.7802	+0.0314	0.0108	*
DeepSurv	0.7879	+0.0237	0.0448	*
